# Impact of scatter correction on personalized dosimetry in selective internal radiotherapy using ^166^Ho-PLLA: a single-center study including Monte-Carlo simulation, phantom and patient imaging

**DOI:** 10.1186/s40658-024-00639-x

**Published:** 2024-04-02

**Authors:** Benoît Collette, Marie Mannie-Corbisier, Ana-Maria Bucalau, Nicolas Pauly, Gontran Verset, Rodrigo Moreno-Reyes, Patrick Flamen, Nicola Trotta

**Affiliations:** 1 Department of Nuclear Medicine, Hôpital Universitaire de Bruxelles (HUB), Université Libre de Bruxelles (ULB), Route de Lennik 808, 1070 Brussels, Belgium; 2 Department of Nuclear Metrology, Brussels School of Engineering, Université Libre de Bruxelles (ULB), Brussels, Belgium; 3 Department of Gastroenterolgy, Hepatopancreatology and Digestive Oncology, Hôpital Universitaire de Bruxelles (HUB), Université Libre de Bruxelles (ULB), Brussels, Belgium; 4 Laboratory of Image Synthesis and Analysis, Brussels School of Engineering, Université Libre de Bruxelles (ULB), Brussels, Belgium

**Keywords:** Holmium-166, Selective internal radiotherapy, Transarterial radioembolization, Personalized dosimetry, Scatter correction, Monte-Carlo

## Abstract

**Background:**

Developments in transarterial radioembolization led to the conception of new microspheres loaded with holmium-166 (^166^Ho). However, due to the complexity of the scatter components in ^166^Ho single photon emission computed tomography (SPECT), questions about image quality and dosimetry are emerging. The aims of this work are to investigate the scatter components and correction methods to propose a suitable solution, and to evaluate the impact on image quality and dosimetry including Monte-Carlo (MC) simulations, phantom, and patient data.

**Methods:**

Dual energy window (DEW) and triple energy window (TEW) methods were investigated for scatter correction purposes and compared using Contrast Recovery Coefficients (CRC) and Contrast to Noise Ratios (CNR). First, MC simulations were carried out to assess all the scatter components in the energy windows used, also to confirm the choice of the parameter needed for the DEW method. Then, MC simulations of acquisitions of a Jaszczak phantom were conducted with conditions mimicking an ideal scatter correction. These simulated projections can be reconstructed and compared with real acquisitions corrected by both methods and then reconstructed. Finally, both methods were applied on patient data and their impact on personalized dosimetry was evaluated.

**Results:**

MC simulations confirmed the use of *k* = 1 for the DEW method. These simulations also confirmed the complexity of scatter components in the main energy window used with a high energy gamma rays component of about half of the total counts detected, together with a negligible X rays component and a negligible presence of fluorescence. CRC and CNR analyses, realized on simulated scatter-free projections of the phantom and on scatter corrected acquisitions of the same phantom, suggested an increased efficiency of the TEW method, even at the price of higher level of noise. Finally, these methods, applied on patient data, showed significant differences in terms of non-tumoral liver absorbed dose, non-tumoral liver fraction under 50 Gy, tumor absorbed dose, and tumor fraction above 150 Gy.

**Conclusions:**

This study demonstrated the impact of scatter correction on personalized dosimetry on patient data. The use of a TEW method is proposed for scatter correction in ^166^Ho SPECT imaging.

## Background

Transarterial radioembolization (TARE) is used for the treatment of non-metastatic liver cancer. This technique of selective internal radiotherapy (SIRT) combines energy deposition due to ß^−^ emission and embolization of blood vessels (arterial feeders) in order to induce tumor necrosis through microspheres loaded with radioisotopes [1]. Recently, a new kind of microspheres emerged: poly-L-lactic acid microspheres loaded with holmium-166, or ^166^Ho-PLLA (QuiremSpheres®, Terumo Europe NV). Smits et al*.* studied their use in chemorefractory liver metastases and showed that TARE was feasible and safe with a maximum whole liver dose tolerance of 60 Gy [2]. In a recent study, Bastiaannet et al*.* demonstrated a dose–response link with a more personalized dosimetry, i.e. a compartmental model [3].

^166^Ho-PLLA microspheres (with a 26.8 h half-life) have a mean diameter of 30 μm (20 to 50 μm) and emit electrons (1.77 MeV maximum with a 48.8% probability and 1.86 MeV maximum with a 49.9% probability), photons (80.6 keV with a 6.7% probability and 1379.4 keV with a 0.9% probability), while being paramagnetic [4]. Direct imaging of their distribution in the human body is therefore possible with gamma-cameras (through the 80.6 keV emission photopeak), even for TARE simulation purposes, and through magnetic resonance imaging (MRI) for post-treatment imaging only. Moreover, Smits et al*.* demonstrated a benefit in the use of a small amount of ^166^Ho-PLLA (250 MBq or approximately 3 million microspheres) for simulation purpose compared to the classical macro aggregated albumin (with technetium-99 m or ^99m^Tc-MAA) simulation in terms of agreement between predictive and post-treatment dosimetry [5]. However, considering the different timing between simulation and treatment, these findings need more studies to be confirmed, including compartmental dosimetry. Therefore, an accurate dosimetry, i.e., a quantitative imaging, requires an accurate scatter correction. Scatter is defined as all the physical contributions artefactually increasing the number of counts in the main acquisition energy window (i.e., noise), whereas attenuation defines all the physical phenomena responsible for decreasing the number of counts in the main window. Attenuation is nowadays easily corrected using attenuation maps generated from computed tomography (CT) acquisitions, as the worldwide spread-out of hybrid SPECT-CT systems made its correction very accessible. Conversely, scatter correction is still challenging, even more in the context of a ^166^Ho SPECT investigation [6].

Ogawa et al*.* classified scatter correction techniques for SPECT imaging based on different methods, including deconvolution, energy-weighted, dual energy window, Monte-Carlo approach for iterative reconstructions, and asymmetrical window [7]. Other approaches are also described in literature as image filtering methods [8]. The critical differences between those methods concern when the correction is applied (before or during reconstruction) and if the inclusion of spatial information is added. This last point is crucial because the ^166^Ho energy spectrum may vary in every pixel due to the sources distribution and the size of the objects, therefore impacting the model of the scatter components. Nevertheless, the simplicity, practicability, and applicability in clinical routine are also factors to be considered. Furthermore, the calculation time or the number of different concurrent images acquired due to manufacturers’ limitations, and, thus, the transferability on existing commercial devices are also to be acknowledged. Dual energy window (DEW) methods are indeed valuable candidates and are widely used. However, in the case of a complex scatter environment as, for example, multiple energy peaks in the energy spectrum, they could provide an inaccurate scatter correction. Therefore, Ogawa et al*.* introduced the triple energy window (TEW) methods, where surrounding scatter contributions at both sides of the main window are considered [7]. This method appears to us to be very promising to improve scatter correction in ^166^Ho SPECT given the shape of a patient energy spectrum [9] and knowing all the complexity of the scatter components. To the best of our knowledge, a detailed investigation in the context of ^166^Ho SPECT has yet to be conducted about this method.

Bayouth et al*.* studied ^166^Ho and scatter correction using DEW methods, customizing the original (general) method introduced by Jaszczak et al*.* [10]. Those authors used a main window centered on 80 keV and a secondary window centered on 120 keV (with a width of 20% of the energy peak value each). The image obtained from counts collected in the secondary window is subtracted to the one linked to the main window after multiplication by a constant *k*. Investigations based on an anthropomorphic phantom data by Bayouth et al*.* led to a *k* = *1* for ^166^Ho scatter correction. This value of *k* is considered as optimal for quantification purposes and is supposed to be constant for every pixel in every projection. This study also highlighted that dead time was significant above 400 MBq, that High Energy collimators are optimal as the only ones ensuring a constant sensitivity between 0 and 35 cm, and that Medium Energy collimators are more suitable than Low Energy collimators for ^166^Ho imaging. Furthermore, it was noted that high energy photons (above 500 keV) pass through all kind of collimator septa [11]. Stella et al*.* also confirmed the dead time above 400 MBq and highlighted the poor spatial resolution [6].

More recently, due to the developments of ^166^Ho-PLLA TARE, other authors investigated and customized scatter correction for ^166^Ho SPECT. Elschot et al*.* clearly discouraged the use of multiple window methods because of the presence of X rays, which would make it impossible to perform an accurate scatter correction [12]. TEW methods were considered inadequate. As the actual reconstruction methods available on the commercial SPECT gamma-cameras do not consider all the scatter components of the spectrum, those authors propose the use of their own method [6]. Nevertheless, their recommendation is not easy to translate on other SPECT systems because of the specific modeling underlying. This method developed by de Wit et al*.* includes a so-called *downscatter* correction based on the old dual energy window method proposed by Bayouth et al*.* [11, 13]: a 12% wide energy window is centered at 118 keV. As already observed, the presence of the bremsstrahlung and high energy photon emissions hugely impact the scatter modeling and their contribution must be considered [12]. de Wit et al*.* combine an attenuation correction, a (down)scatter correction and a Monte-Carlo based approach. The use of TEW methods is again not recommended due to the supposed presence of X rays near 80.6 keV [13]. Interestingly, the influence of the downscatter on acquired attenuation maps on SPECT/CT systems is here negligible [13].

The aims of this work are to investigate scatter components and correction methods to propose a suitable solution that can also be convenient and easily implemented on SPECT systems from different manufacturers. Moreover, we evaluate the impact on image quality and dosimetry including Monte-Carlo simulations, phantom, and patient data. Therefore, we focused on the potential and the applicability of a TEW method in the context of ^166^Ho SPECT, compared to the well-documented DEW method. To the best of our knowledge, this is the first study investigating all the scatter components by Monte-Carlo simulations in the field of ^166^Ho SPECT imaging (including the X rays contribution), and quantifying the impact of those methods on personalized dosimetry.

## Methods

### Phantom

**Fig. 1:**
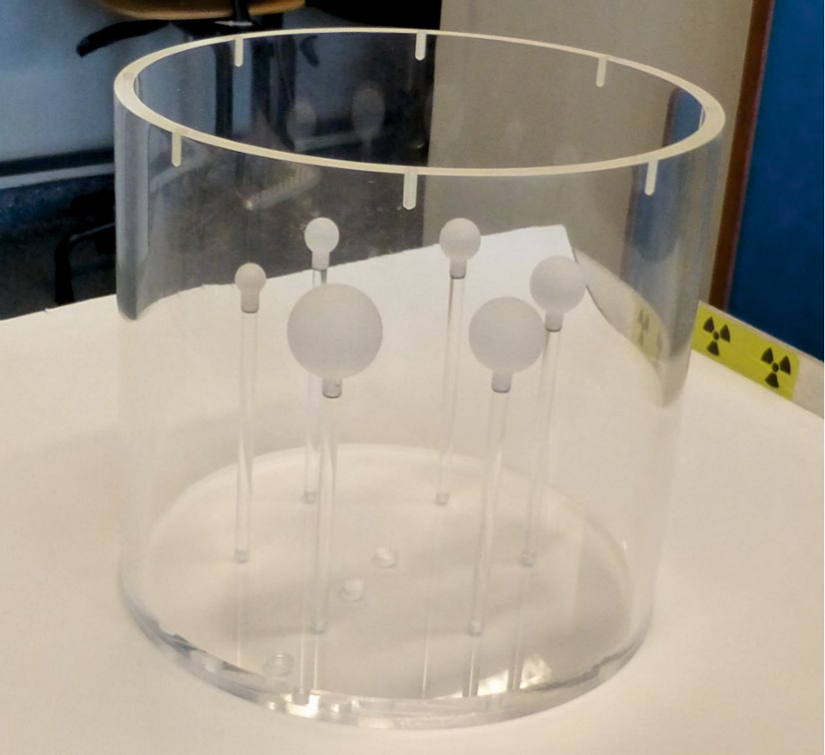
The Jaszczak Pro-NM Performance, complying with NEMA standards publication (NU 1-2001), cold rods and cold spheres inserts being removed and replaced by fillable spheres

The Jaszczak Pro-NM Performance, complying with National Electrical Manufacturers Association (NEMA) standards publication (NU 1-2001), consists of a cylindrical phantom with an inside cylinder diameter of 206 mm, an inside cylinder height of 186 mm, and a cylinder wall thickness of 7 mm. The cold rods and cold spheres inserts were removed. Instead, a complementary set of fillable spheres was used (with inner diameters of 9.9 mm, 12.4 mm, 15.4 mm, 19.8 mm, 24.8 mm, and 31.3 mm, and a wall thickness of 2 mm), as illustrated on the Fig. 1.

The phantom and the six spheres were filled with a holmium-166 chloride (^166^HoCl) solution mixed with Diethylene Triamine Penta Acetic (DTPA) acid to avoid inhomogeneous distribution of the radioisotope due to stickiness to the plastic walls of the phantom and specifically of the spheres. The activity ratio between spheres and background was 18:1. The goal was to respect the well-known limit of 400 MBq [6], [11] for the total activity, and to approach the real activity used for a typical ^166^Ho-PLLA simulation when 50–200 MBq are administered to the patient at our institution in a typical tumor volume of 100 ml (calculated as the median size of hepatocellular carcinomas (HCC) treated with ^166^Ho-PLLA in our center). We finally filled the hollow spheres with 29 MBq homogeneously distributed in the 31.5 ml of the total spheres volume, to approach a 1 MBq/ml ratio mimicking what was experienced with tumors, and filled the background with 312 MBq homogeneously distributed in the 6200 ml of the total background volume, for a total of 341 MBq at the beginning of the first acquisition. The phantom was acquired 4 times to a final total activity of 321.5 MBq at the beginning of the last acquisition, therefore staying under the recommended 400 MBq [6], [11].

### Patients

Nineteen patients have been included, for a total of 21 treatments, 2 patients being treated twice. All the patients except one (cholangiocarcinoma), presented unresectable hepatocellular carcinoma (HCC), diagnosed according to European Association for the Study of the Liver and European Organisation for Research and Treatment of Cancer (EASL-EORTC) guidelines [14]. Our workflow was performed over two separate sessions: the simulation and the treatment (TARE).

Simulation evaluation started with an angiography in order to obtain a precise map of the patients’ abdominal vascular anatomy. Then, the simulation included the administration of 40 to 179 MBq of ^166^Ho-PLLA, so called *“scout”* (QuiremScout®), following the tumor size and perfusion, in order to predict the distribution pattern expected after treatment. ^166^Ho-PLLA TARE was performed (with QuiremSpheres®) within 14 days after the simulation. The amount of ^166^Ho activity administered to a patient for TARE purposes depended on the tumor perfusion and the predicted tumor absorbed dose. As previously mentioned, recent data (not focusing on HCC) showed a dose–response link, i.e. an estimated tumor dose of 168 Gy needed for a partial response and of 232 Gy for a total response [3]. Following these requirements, the compartmental dosimetry software Q-Suite™ 2.0, was used to calculate the activity that needed to be injected, in order to expect tumor doses between 169 and 300 Gy for the 21 treatments, leading to administered activities from 1.86 to 13 GBq. The absorbed dose to the lung never exceeded 30 Gy in a single treatment or 50 Gy in multiple treatments.

One post-treatment dosimetry was excluded because the threshold of 400 MBq [6], [11] was overcome during the imaging, leading to a final dataset of 21 simulations and 20 treatments.

### Imaging and reconstruction

The activity uptake is visualized by a whole body planar imaging and SPECT imaging of the abdomen, including a low dose computed tomography (CT).

Imaging was performed using the same Philips BrightView XCT SPECT/CT system (Philips Medical Systems, Cleveland, Ohio, USA) for every session. SPECT/CT acquisitions of the phantom were realized with the same acquisition parameters used for the SPECT/CT imaging of patients, except the distance from collimators being fixed at 30 cm from the phantom center. This distance was as close as possible for patients (using autobody contouring).

**Fig. 2:**
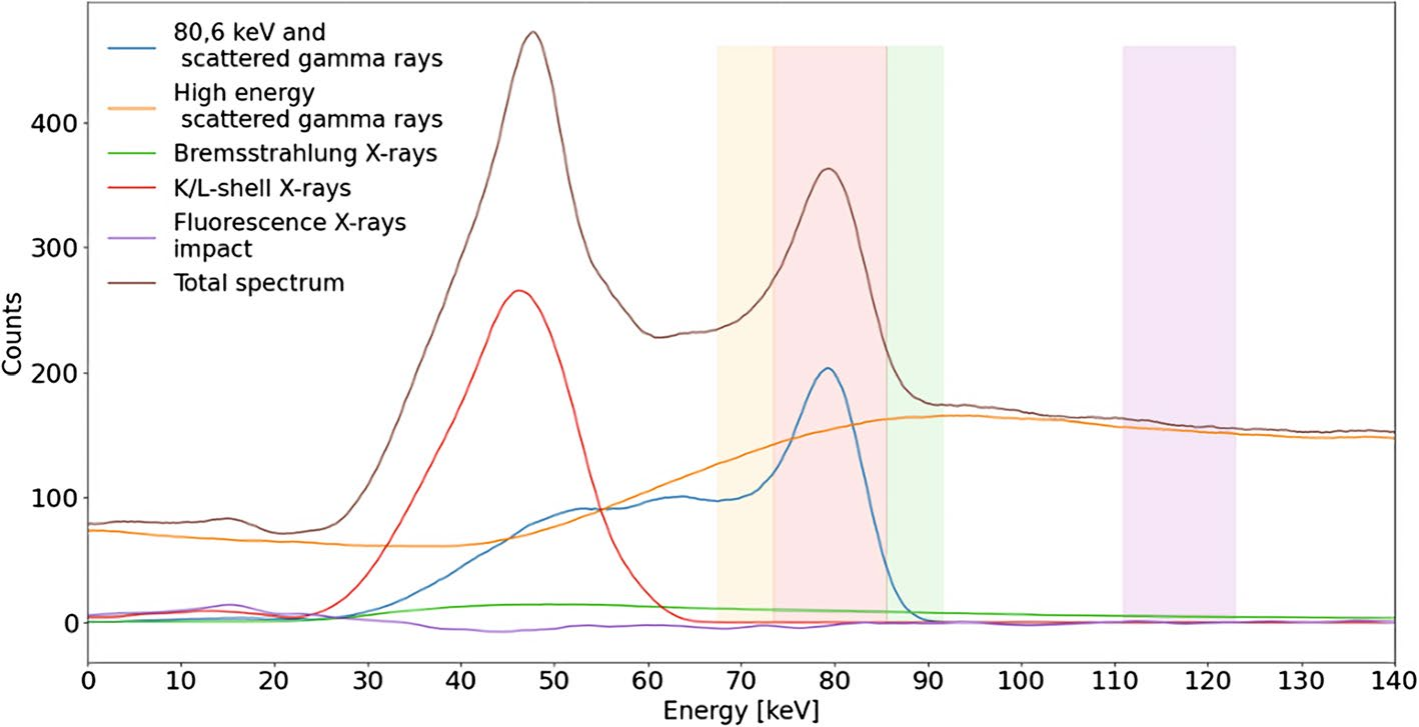
Decomposed energy spectrum simulated using GATE. Spherical source in cylindrical water phantom with MEGP collimator. The yellow energy window 1 (W1) is 6 keV wide, the red energy window 2 (W2) is 12 keV wide, the green energy window 3 (W3) is 6 keV wide, and the purple energy window 4 (W4) is 12 keV wide

The thicker collimator available was used: the Medium Energy General Purpose (MEGP) collimator, with 0.86 mm septa thickness of lead and a length of 58.4 mm [15]. This was the best possible choice knowing its intrinsic limitations studied by Bayouth et al*.* and reminded in the introduction [11]. A main acquisition energy window around 80.6 keV with 15% width (equivalent to 12 keV) was chosen [4] (*“window 2”* or *“W2”* in Table 1). For patients’ acquisitions, firstly a whole-body scan was acquired to assess the absence of extra hepatic deposition (18 cm/min, 256 pixels wide). Then, a SPECT imaging (lung-liver centered) was performed to estimate the spatial distribution of ^166^Ho-PLLA (120 projections, 30 s/projection, 360°, 128 × 128 pixels matrix size) [4], together with a cone-beam (CB) CT imaging (for attenuation correction). Concerning the scatter correction, 2 extra acquisition energy windows were set, the camera being limited to 3 concurrent imaging. The first one was set around 118 keV [4, 11, 13] with 10,2% width (12 keV) for DEW method purposes (*“window 4”* or *“W4”* in Table 1). We conceived an alternative window, corresponding to the sum of 2 narrow windows around the main window, which are then considered as a unique acquisition window: 71.56 keV with 8.39% width (6 keV) (*“window 1”* or *“W1”* in Table 1) and 89.65 keV with 6.69% width (6 keV) (*“window 3”* or *“W3”* in Table 1). The latter acquisition window (W1 + W3) was used for the TEW method correction. Every count collected in one of those 3 acquisition windows (W2, W1 + W3 and W4) lead to a different image. The sizes of those acquisition energy windows (illustrated on the Fig. 2) were obviously chosen to easily apply the scatter correction by a user-friendly mathematical operation consisting in the subtraction of the acquisition image (projection) corresponding to one of the extra windows from the acquisition image (projection) corresponding to the main window.

**Table 1:** Energy window values

Window	Lower limit (keV)	Upper limit (keV)	Center and width
W1	68.56	74.56	71.56 keV ± 8.39%
W2	74.56	86.65	80.6 keV ± 15.0%
W3	86.65	92.65	89.65 keV ± 6.69%
W4	112.0	124.0	118.0 keV ± 10.2%

Every projection image (uncorrected, DEW-corrected, TEW-corrected, or numerically simulated) used in this work was reconstructed with the same method using the Ordered Subset Expectation Maximization (OSEM) algorithm with 10 iterations, and 8 subsets. An additional Butterworth filter (cutoff = 0.25 and order = 1.5) was applied for visualization purpose.

### Numerical simulation

The numerical simulations were performed using the GATE 7.0 software which is an open-source simulation software for medical imaging and radiotherapy purposes [16]. It has been developed by the international OpenGATE collaboration which regroups 18 worldwide institutions. GATE uses the well-known Geant4 software to simulate the particles production and transport. It can manage by itself the detector and signal-processing chain. The particle transport is based on a Monte-Carlo method which allows to reproduce numerically the underlying physics that are the cause of the corresponding image output from a SPECT acquisition. In practice, firstly, the different components of the SPECT device used in this work were reproduced in GATE considering their material and shape, adapted from the script from OpenGATE collaboration and University Hospital Carl Gustav Carus in Dresden [17]. The Jaszczak Pro-NM Performance phantom was also coded in GATE. Then, the physical interactions of interest were activated considering for this purpose a full standard physics list (available in Geant4).

At the end of the simulation process, we obtained two major outputs from GATE: first, a characterization of the spectrum components considering real acquisition conditions for a sphere centered in a water cylinder mimicking the size of the Jaszczak phantom, and second, a Jaszczak phantom scatter-free and attenuation-free projections set considering a vacuum environment in place of real matter for phantom and air.

### Quality assessment

To compare scatter correction methods, we chose to evaluate Contrast Recovery Coefficients (CRC) on Jaszczak phantom reconstructed images. CRC were calculated for a sphere *i* following the Eq. 1 [18]:

1 $${CRC}_{i}=\frac{{pixcounts}_{{sphere}_{i}}/{activity}_{{sphere}_{i}}}{{pixcounts}_{background}/{activity}_{background}}$$

The volume of interest (VOI) for a sphere is defined as all the voxels inside the physical (CT) volume avoiding border voxels affected by partial volume effect. The background VOI is a cylinder (with a 25 mm radius) centered in the transverse plan which includes the centers of the Jaszczak spheres. As explained by Stam et al*.* [18], *pixcounts*_*sphere,i*_ represents the activity concentration measured on the image in sphere *i*, *activity*_*sphere,i*_ represents the actual activity concentration in sphere *i*, *pixcounts*_*background*_ is the activity concentration measured on the image in the background volume, and *activity*_*background*_ is the actual activity concentration in the background volume.

The calculated CRC were fitted using a sigmoid interpolation [19] following the Eq. 2:

2 $$S\left(x\right)=\frac{a}{1+{e}^{-b.(x-c)}}-d$$

In this equation, *a* = *1* because CRC tends to 1 after a certain sphere volume, as showed by Cherry et al*.* [19]. The other parameters are determined using a least square method. The variable *x* is the ratio sphere diameter / Full Width at Half Maximum (FWHM) reflecting the spatial resolution.

To compare scatter correction methods, we also evaluated Contrast to Noise Ratio (CNR) on Jaszczak phantom reconstructed images. The CNR for a sphere *j* is calculated following the Eq. 3 [20]:

3 $${CNR}_{j}=\left|\frac{{C}_{{sphere}_{j}}-{C}_{background}}{{\sigma }_{background}}\right|$$

As explained by van Gils et al*.* [20], *C*_*sphere,j*_ is the average number of counts in the sphere *j*, *C*_*background*_ is the average number of counts in the background VOI and *σ*_*background*_ is the standard deviation in the background VOI.

Other metrics such as the homogeneity and/or coefficient of variation of axial and radial profiles are available, but those metrics are better suited to a homogeneous phantom, which was not what was used in our study.

### Dosimetry

The compartmental dosimetry software Q-Suite™ 2.0 was used to assess predictive dosimetry and post-treatment dosimetry for every treatment. For predictive dosimetry only, Q-Suite™ 2.0 at first predicts the lung dose after contouring the lungs and the whole liver on the CT images associated to the SPECT and specifying the planned activity to be administered. This method is indeed a volumetric SPECT-CT evaluation. Then, CT, T1- or T2-weighted MRI can be used to define compartments in the liver, as tumors and non-tumoral liver (NTL) tissue. A manual rigid registration is available to co-register the SPECT-CT images used to generate the dose map.

In Q-Suite™ 2.0, Dose Point Kernel model [21] is only available for post-treatment purposes, whereas Local Dose Deposition model [22] is available for predictive and post-treatment dosimetry. We therefore used the latter model for both dosimetries.

The TEW method was chosen to correct the simulation images used to calculate (in a predictive way) the activity to administer for TARE treatments.

### Statistical analysis

To evaluate the impact of the choice of the scatter correction on personalized dosimetry, we use paired *t*-tests between parameters’ distributions obtained for the 21 pre-treatment dosimetries conducted on DEW and TEW method corrected images, as well as for the 20 post-treatment dosimetries also realized on DEW and TEW method corrected images. The parameters taken into account are the following outputs of Q-Suite™ 2.0: tumor dose, non-tumoral liver dose, tumor fraction receiving at least 150 Gy, and non-tumoral liver fraction receiving between 0 and 50 Gy. These analyses led us to 8 statistical comparisons of 16 datasets.

## Results

GATE 7.0 software allowed us to obtain the decomposed spectrum from the spherical source of ^166^Ho in a cylindrical water phantom shown on Fig. 2. All the different components are present in the range 0–140 keV: the 80.6 keV peak and his scattered gamma rays spectrum, the high energy scattered gamma rays spectrum, the bremsstrahlung X rays spectrum, the K/L-shell X rays spectrum and its peak around 49 keV, the fluorescence X rays impact, and the total spectrum as seen from a gamma-camera acquisition.

Table 2 shows the relative quantification of the 4 main energy spectrum components in the full energy range and the energy windows used for acquisition and scatter correction (W1 to W4).

**Table 2:** Relative quantification of the energy spectrum components in full simulated energy range and energy windows used for scatter correction

Window	80.6 keV gamma rays and associated scatter (%)	High energy scattered gamma rays (%)	X rays peak (%)	Bremsstrahlung X rays (%)
Full range	6.1	86.6	5.6	1.7
W1	41.5	54.4	0.0	4.4
W2	48.3	48.7	0.0	3.0
W3	6.5	88.9	0.0	4.4
W4	0.0	96.9	0.0	3.1

**Fig. 3:**
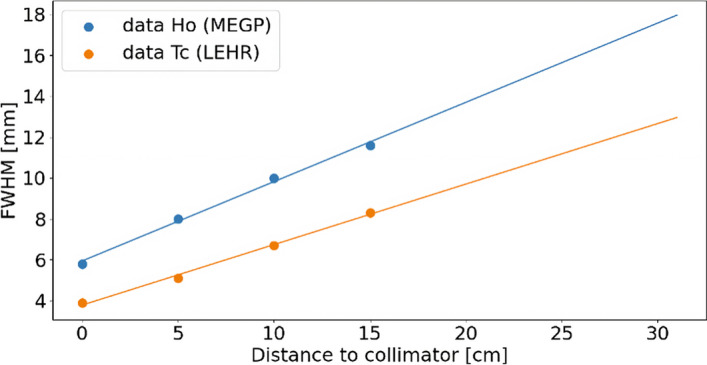
Spatial resolution of the gamma-camera. Both heads’ mean spatial resolution comparison between ^99m^Tc line-source in air with LEHR collimator and ^166^Ho line-source in air with MEGP collimator on our Philips BrightView XCT

For CRC and CNR comparisons, we choose to plot our results following an X axis labeled in *“Sphere diameter/FWHM”* units [19], where FWHM is the Full Width at Half Maximum reflecting the spatial resolution and coming from both heads mean measurements showed in the Fig. 3. In this figure, ^166^Ho imaging with a MEGP collimator is compared to a more classical configuration of ^99m^Tc imaging with Low Energy High Resolution (LEHR) collimator. The ^166^Ho imaging with MEGP collimator led to an increased FWHM for all the distances. Those spatial resolution measurements are obtained following national guidelines using a capillary tube placed at several distances from collimators.

**Fig. 4:**
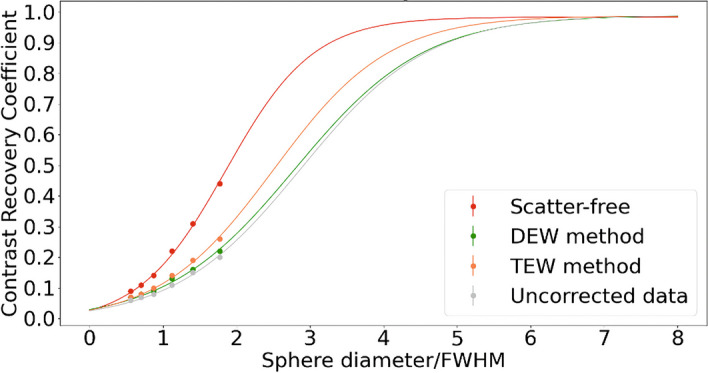
Contrast Recovery Coefficients. Contrast recovery coefficients and sigmoid fitting from Jaszczak real acquisitions and numerical simulations using GATE. The red points and curve fitting are quantified on scatter-free images from numerical simulations, the green and orange points and curves fittings are quantified on scatter-corrected images from real acquisitions, and the gray points and curve fitting are quantified on uncorrected images from real acquisitions

**Fig. 5:**
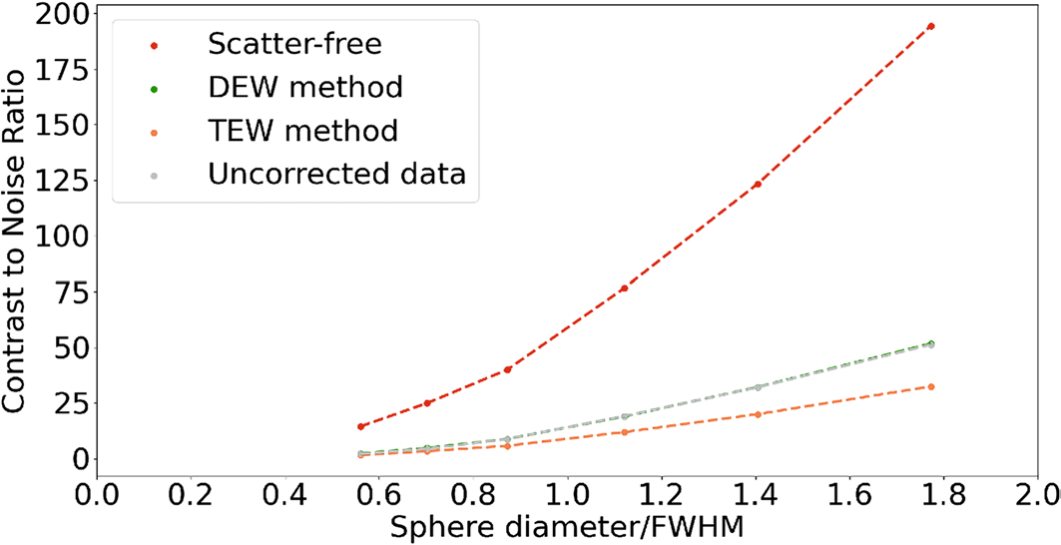
Contrast to Noise Ratios. Contrast to noise ratios from Jaszczak real acquisitions and numerical simulations using GATE. The red points and dotted lines are quantified on scatter-free images from numerical simulations, the green and orange points and dotted lines are quantified on scatter-corrected images from real acquisitions, and the gray points and dotted lines are quantified on uncorrected images from real acquisitions. The dotted lines only represent visual connections

Thanks to GATE simulations, we obtained Jaszczak phantom scatter-free and attenuation-free projections considering a vacuum environment in place of real matter for phantom and air, leading to the CRC comparison shown in Fig. 4 and the CNR comparison shown in Fig. 5. CRC and CNR were also quantified from Jaszczak reconstructions of DEW method corrected, TEW method corrected and uncorrected (for scatter only) projections, all those projections being corrected for attenuation through CT attenuation map. As visible in Fig. 4, the curve interpolating scatter-free data is above all other curves and reach the horizontal asymptote between 4 and 5 sphere diameters / FWHM. The TEW method curve is the closest to the scatter-free curve and reach the horizontal asymptote near 6 sphere diameters / FWHM. The DEW method curve and the curve interpolating the uncorrected data are above and close to each other, reaching the horizontal asymptote near 7 sphere diameters / FWHM. In the Fig. 5, the plotted scatter-free values are also clearly higher than all the others for every sphere diameter/FWHM. The DEW method is superposed to the uncorrected data and not distinguishable, with relative differences varying from 1 to 8%. The plotted TEW method values are lower than all the others and present a relative difference with uncorrected data values ranging from 24 to 38%.

The results of our statistical analysis, using Q-Suite™ 2.0 for dosimetry purposes, are shown in Tables 2 and 3. Regarding those statistical comparisons for 21 pre-treatment (simulation) dosimetries (Table 3) and for 20 post-treatment dosimetries (Table 4), *p*-values (*p*-val) are obtained from paired *t*-test analysis and considered significant if ≤ 0,05. In Table 3, all the *p*-values are significant. In Table 4, only *p*-values regarding the tumor dose and the non-tumoral liver fraction receiving between 0 and 50 Gy are significant. In Table 3, the mean tumor dose evaluated after TEW method correction is 14% higher than the mean tumor dose evaluated after DEW method correction for roughly the same standard deviation. The fraction of tumor receiving at least 150 Gy and the fraction of NTL receiving between 0 and 50 Gy follow both the same trend (8.5% and 5% of mean difference respectively), but the NTL dose doesn’t. In Table 4, data showed an opposite behavior. The mean tumor dose evaluated after TEW method correction is 6% lower than the mean tumor dose evaluated after DEW method correction for roughly the same standard deviation. Both the fraction of tumor receiving at least 150 Gy and the fraction of NTL receiving between 0 and 50 Gy follow the same trend (13% and 3% of mean difference respectively), whereas the NTL dose shows an opposed trend.

**Table 3:** Statistical comparison for 21 pre-treatment (simulation) dosimetries regarding tumor dose, non-tumoral liver (NTL) dose, tumor fraction receiving at least 150 Gy, and non-tumoral liver (NTL) fraction receiving between 0 and 50 Gy

Criteria	DEW [mean ± std dev]	TEW [mean ± std dev]	p-val
Tumor dose	201.86 ± 49.73 Gy	229.43 ± 44.30 Gy	0.01*
NTL dose	40.00 ± 22.34 Gy	36.90 ± 23.47 Gy	0.02*
≥ 150 Gy tumor fraction	72.57 ± 23.55%	81.17 ± 17.17%	0.03*
0–50 Gy NTL fraction	71.30 ± 16.27%	75.60 ± 16.21%	0.01*

p-values (p-val) are obtained from paired t-test analysis and considered significant if ≤ 0.05 (*)

**Table 4:** Statistical comparison for 20 post-treatment dosimetries regarding tumor dose, non-tumoral liver (NTL) dose, tumor fraction receiving at least 150 Gy, and non-tumoral liver (NTL) fraction receiving between 0 and 50 Gy

Criteria	DEW [mean ± std dev]	TEW [mean ± std dev]	p-val
Tumor dose	178.95 ± 63.82 Gy	168.30 ± 64.13 Gy	0.04*
NTL dose	34.90 ± 20.70 Gy	35.20 ± 21.06 Gy	0.59
≥ 150 Gy tumor fraction	57.61 ± 29.60%	50.88 ± 31.11%	0.07
0–50 Gy NTL fraction	75.95 ± 17.78%	73.64 ± 17.93%	0.04*

p-values (p-val) are obtained from paired t-test analysis and considered significant if ≤ 0.05 (*)

## Discussion

The aims of this work are to investigate scatter components and correction methods to propose a suitable and transferrable solution for SPECT systems, and to evaluate the impact of this choice on image quality and dosimetry including Monte-Carlo simulations, phantom, and patient data.

Our data (Fig. 2 and Table 2) showed that photons’ count in W4 is well representative of the high scattered gamma rays (96.9%). Comparing the area under the high energy scattered gamma rays curve between W2 and W4, they confirm that *k* = 1 is an appropriate choice for the DEW method, as Bayouth et al*.* suggested [10]. However, this method supposes that the high energy scattered gamma rays component is the only scatter contribution, therefore ignoring to take into account the 80.6 keV scattered gamma rays in evidence in Fig. 2. Moreover, Buvat et al*.* [8] suggested that such a difference in terms of energy between high energy scattered gamma rays detected in W2 and W4 means more energy loss for gamma rays from W2, thus more length path in the matter. The question of the appropriate subtraction of photons’ count in a pixel, knowing that their origins are very different, is crucial. Indeed, this uncertainty justifies the need to evolve towards a more accurate method of correction.

From the analysis of the ^166^Ho spectrum (represented in Fig. 2 and quantified in Table 2), we concluded that the X rays contribution from W1 to W4 can be considered as negligible, which is in contrast with results showed in previous works [12]. Indeed, the most important scatter contribution in W2 comes from high energy scattered gamma rays (48.7% of the total counts acquired in the main window) but also from 80.6 keV scattered gamma rays (48.3%). Bremsstrahlung X rays represents only a small part (3%) while fluorescence X rays have no impact at all.

It is worth to note that these results depend on the type and the thickness of the material around the ^166^Ho source. In this part of our work, the water and plexiglass thickness are constant in all directions due to the cylindrical shape of the Jaszczak phantom simulated.

Our results can be compared to those of Elschot et al*.* [12]. With respectively a 40 × 40 × 2 cm^3^ PolyMethyl MethAcrylate (PMMA) layer in front of the source (for planar line source imaging with Monte Carlo N Particle eXtended (MCNPX) simulation), they found a count repartition of 50.7%, 45.2%, and 4.1%, for the 80.6 keV photopeak gamma rays, high energy scattered gamma rays, and bremsstrahlung X rays, respectively, whereas using a 40 × 40 × 20 cm^3^ PMMA layer, the repartition was 30.8%, 64.2%, and 5%, respectively. The contribution of the high energy gamma rays in the main window increases with the thickness while the 80.6 keV photopeak gamma rays contribution decreases.

Taking our spectrum study into account, the TEW method seems promising. Its added value is shown in Fig. 4, where we compared it with the DEW method by CRC evaluation. We can clearly see that the DEW method curve is close to the uncorrected data curve, while the TEW method curve is closer to the scatter-free curve.

On the other hand, in Fig. 5, we see that the TEW method seems to generate a higher level of noise than the DEW method, as the latter being very close to the uncorrected data with an apparent lacking noise addition. This increased noise could be linked to the size (6 keV) of the adjacent windows (W1 and W3) used for the TEW method in this work. A larger window size could generate less noise, unfortunately we were not able to investigate this link due to the limitations of our gamma-camera. Nevertheless, this impact should be investigated in future works on SPECT systems allowing to set enough energy windows, which is generally limited for clinical purposes, or enough concurrent imaging where the acquisition duration or the number of counts would be adapted regarding the window size.

**Fig. 6:**
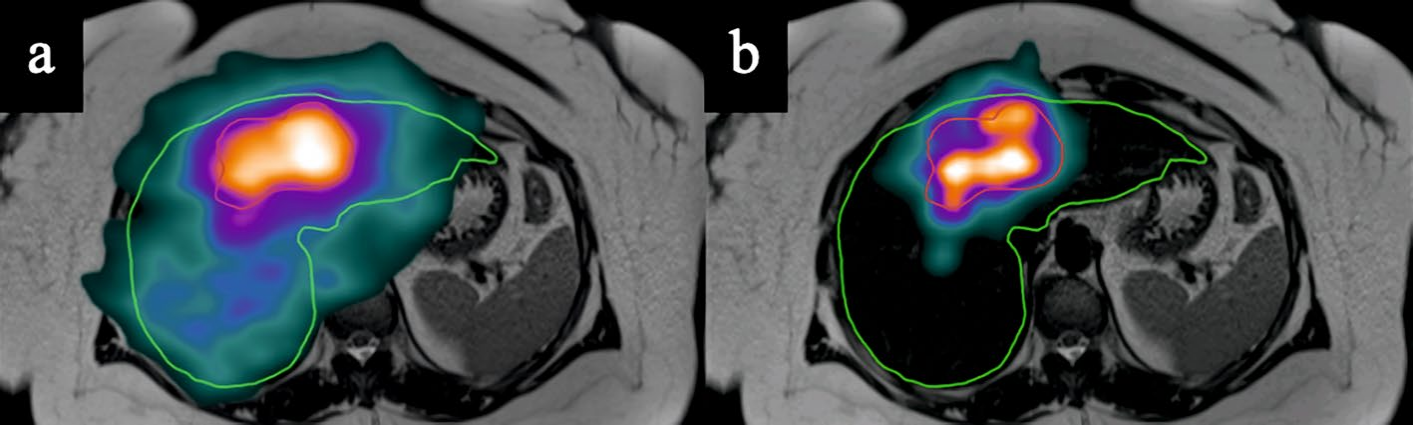
^166^Ho-PLLA SPECT/CT on MRI images (T2 sequence) for simulation purposes in a 50-years old woman suffering from cholangiocarcinoma through segments II, Va and VIII, DEW method being applied on the left image (**a**) and TEW method on the right (**b**), tumor in red

**Fig. 7:**
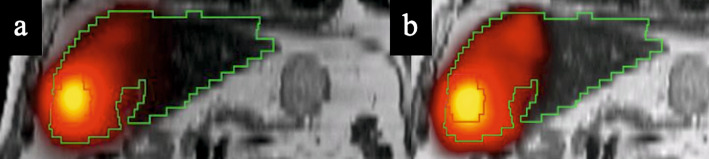
^166^Ho-PLLA SPECT/CT on MRI images (T2 sequence) for post-treatment dosimetry in a 84-years old man suffering from hepatocellular carcinoma through segment IVb, DEW method being applied on the left image (**a**) and TEW method on the right (**b**), tumor in red

The TEW method still does not allows to reach the perfect scatter correction, but it is convenient and it can easily be implemented in clinical routine, which was one of our criteria of choice. Its impact on image quality is already clearly visible on different patient images. Figure 6 shows ^166^Ho-PLLA SPECT/CT on MRI images (T2 sequence) for simulation purposes. The TEW method reduces the scatter and increases the spatial resolution inside the tumor, making the intra-tumoral activity (and dose) repartition more accurate. Figure 7 shows ^166^Ho-PLLA SPECT/CT on MRI images (T2 sequence) for post-treatment dosimetry. The TEW method here is useful to assess extra-tumoral activity (and dose) deposition, not clearly noticeable with the DEW method.

**Fig. 8:**
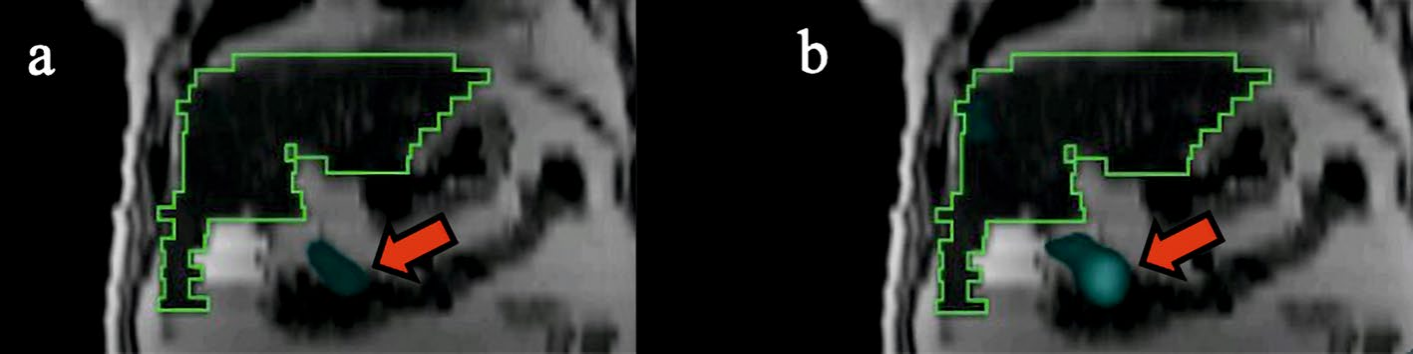
^166^Ho-PLLA SPECT/CT on MRI images (T2 sequence) for simulation purposes in a 71-years old man suffering from hepatocellular carcinoma through segment I, VI and VII, DEW method being applied on the left image (**a**) and TEW method on the right (**b**). The red arrow shows the intestinal uptake

Regarding the safety of the treatment, there is a critical need to assess extra-hepatic activity deposition during pre-treatment planning, or simulation. Clinically, one of the major exclusion criteria consists in the possible activity that can be found outside the liver and the lung, as an intestinal uptake. Usually, physicians only realize a visual evaluation of this intestinal uptake, because no specific limit exists in terms of counts or activity. Figure 8 shows ^166^Ho-PLLA SPECT/CT on MRI images (T2 sequence) for simulation purposes. The intestinal activity deposition (the red arrow on Fig. 8) is difficult to assess using DEW method corrected images, while it is visually striking on the images corrected with the TEW method.

Moreover, the choice of the scatter correction method for SPECT imaging in the context of a TARE conducted with ^166^Ho-PLLA microsphere has a significant impact on dosimetry, as reported in Tables 2 and 3.

Table 3 shows that *p*-values are significant for all the criteria evaluated for predictive pre-treatment dosimetry (simulation). Table 4 shows that *p*-values are significant for 2 out of the 4 criteria evaluated for the post-treatment evaluation: tumor dose and the non-tumoral liver fraction receiving between 0 and 50 Gy. Nonetheless, those 2 parameters are usually critical for the efficacy and the safety evaluation of the treatment. Moreover, the *p*-value for the tumor fraction receiving at least 150 Gy is not significant but still close to the threshold. Finally, about the non-tumoral liver dose, a significant difference is more difficult to obtain because, by considering extended volumes, small differences in terms of administered activity make negligible differences in terms of calculated dose.

The trends observed in terms of mean differences between results for the DEW method and the TEW method (in Tables 2 and 3), going in opposite ways regarding tumor dose and NTL dose, can be representative of our experience in terms of correlation between pre-treatment (simulation) and post-treatment dosimetries. Our study is designed to use a more personalized dosimetry than previous studies, and our treatments to be as selective as possible. However, we noticed a discrepancy from predictive to post-treatment data leading to an underdosage of the tumor although the predictive personalized dosimetry and the response to the treatment were satisfying, with objective response in all the targeted lesions at three months evaluation. We speculate that the reason is to be linked to an embolic effect due to the use of ^166^Ho-PLLA for simulation purposes. In fact, the predictive versus post-treatment mean difference is exacerbated regarding the tumor dose using the TEW method compared to the DEW method (27% versus 11% respectively), but less evident regarding NTL dose (5% versus 13% respectively). This is probably because the TEW method generates images with a better spatial resolution, less smoothed than the DEW method ones.

The choice of the 4 criteria (the tumor dose, the non-tumoral liver dose, the tumor fraction receiving at least 150 Gy, and the non-tumoral liver fraction receiving between 0 and 50 Gy) for statistical analyses was made due to the recent data showing improved efficacy of a more personalized approach [23], meaning that the appreciation of the tumor dose and the non-tumoral liver dose is crucial for this aim. Furthermore, the intra-tumoral dose repartition is also important because tissue necrosis could be present. Finally, the preserved (or remnant) non-tumoral liver fraction and function should also be considered as important safety indexes, that may be evaluated in association with ^99m^Tc-mebrofenin hepato-biliary scintigraphy SPECT/CT [24].

## Conclusions

The scatter components in ^166^Ho SPECT imaging need a more accurate method to be corrected than the traditional DEW method, which we showed to lead to results close to uncorrected data in terms of CRC. In this context, the TEW method seems promising, still not permitting a perfect scatter correction. The issue concerning the apparent increase of noise level may be linked to the size of the energy windows used and therefore needs further investigation. Nevertheless, we demonstrate in this work its impact on personalized dosimetry for patient undergoing a TARE with ^166^Ho-PLLA microspheres, compared to the DEW method. The TEW method for scatter correction is convenient and easy to be implemented on different SPECT systems for clinical routine.

## Data Availability

The datasets used and/or analysed during the current study are available from the corresponding author on reasonable request.

## Abbreviations

CB: Cone-beam
CNR: Contrast to Noise Ratio
CRC: Contrast Recovery Coefficient
CT: Computed tomography
DEW: Dual energy window
DTPA: Diethylene Triamine Penta Acetic
EASL: European Association for the Study of the Liver
EORTC: European Organisation for Research and Treatment of Cancer
FWHM: Full Width at Half Maximum
HCC: Hepatocellular carcinoma
^166^Ho: Holmium-166
^166^HoCl: Holmium-166 chloride
LEHR: Low Energy High Resolution
MAA: Macro aggregated albumin
MC: Monte-Carlo
MCNPX: Monte Carlo N Particle eXtended
MEGP: Medium Energy General Purpose
MRI: Magnetic resonance imaging
NEMA: National Electrical Manufacturers Association
NTL: Non-tumoral liver
OSEM: Ordered Subset Expectation Maximization
PLLA: Poly-L-lactic acid
PMMA: PolyMethyl MethAcrylate
SIRT: Selective internal radiotherapy
SPECT: Single photon emission computed tomography
TARE: Transarterial radioembolization
^99m^Tc: Technetium-99m
TEW: Triple energy window
VOI: Volume of interest
